# Bioapplications of Cell-SELEX-Generated Aptamers in Cancer Diagnostics, Therapeutics, Theranostics and Biomarker Discovery: A Comprehensive Review

**DOI:** 10.3390/cancers10020047

**Published:** 2018-02-09

**Authors:** Xuehui Pang, Cheng Cui, Shuo Wan, Ying Jiang, Liangliang Zhang, Lian Xia, Long Li, Xiaowei Li, Weihong Tan

**Affiliations:** 1 Key Laboratory of Interfacial Reaction & Sensing Analysis in Universities of Shandong, School of Chemistry and Chemical Engineering, University of Jinan, Jinan 250022, China; pxh1118@yeah.net; 2 Center for Research at the Bio/Nano Interface, Department of Chemistry and Department of Physiology and Functional Genomics, UF Genetics Institute and McKnight Brain Institute, Shands Cancer Center, University of Florida, Gainesville, FL 32611-7200, USA; cuichengcuicheng@gmail.com (C.C.); shuowan@chem.ufl.edu (S.W.); ying214@chem.ufl.edu (Y.J.); lilong@chem.ufl.edu (L.L.); xli147@chem.ufl.edu (X.L.); 3 State Key Laboratory for the Chemistry and Molecular Engineering of Medicinal Resources, School of Chemistry and Pharmaceutical Sciences, Guangxi Normal University, Guilin 541004, China; liangzhang319@163.com; 4 College of Chemistry and Chemical Engineering, Qufu Normal University, Qufu 273165, China; xialian01@163.com; 5 Molecular Science and Biomedicine Laboratory, State Key Laboratory of Chemo/Biosensing and Chemometrics, College of Biology, College of Chemistry and Chemical Engineering, Hunan University, Changsha 410082, China

**Keywords:** cell-SELEX, aptamer, cancer, imaging, drug delivery, therapy, biomarker

## Abstract

Currently, functional single-stranded oligonucleotide probes, termed aptamers, generated by an iterative technology, Systematic Evolution of Ligands by Exponential Enrichment (SELEX), are utilized to selectively target molecules or cells with high affinity. Aptamers hold considerable promise as multifunctional molecules or conjugates for challenging nanotechnologies or bioapplications now and in the future. In this review, we first describe recent endeavors to select aptamers towards live cancer cells via cell-SELEX. We then introduce several characteristic applications of selected aptamers, especially in imaging, drug delivery and therapy. In part, these advances have been made possible via synthesis of aptamer-based nanomaterials, which, by their sizes, shapes, and physicochemical properties, allow such aptamer-nanomaterial complexes to function as signal reporters or drug carriers. We also describe how these aptamer-based molecular tools contribute to cancer biomarker discovery through high-affinity recognition of membrane protein receptors.

## 1. Elucidation

Aptamers, sometimes called “chemical antibodies”, are single-stranded DNA or RNA oligonucleotides commonly containing 20~100 nucleotides. However, aptamer molecules and antibodies are very different in terms of properties and structure [1,2,3,4,5]: (1) antibodies are produced in vivo in a living organism, while aptamers may be developed in vitro by selection and chemical synthesis, allowing easy, fast, and economical reproducibility in different batches; (2) aptamers can be easily conjugated with a series of functional groups or functionalized with different substrates [6] to achieve various diagnostic and therapeutic objectives, with additional advantages of reversible denaturation ability [7,8] and long-term stability in solution or as dry powder; (3) aptamers can bind with their targets with remarkable affinity (*K*_d_ ≈ µM to pM range) by the ability to fold into secondary or tertiary structures. Aptamers can specifically recognize their targets, including, but not limited to, metal ions, nanomaterials, molecules, chemical linkers, fluorophores, as well as proteins and intact cells [9,10], with selectivity comparable to that of antibodies; (4) aptamers are, moreover, nontoxic and exhibit rapid tissue penetration, with little immunogenicity compared to antibodies. These desirable features make aptamers ideal and valuable tools in many fields, such as molecular medicine, especially cancer diagnostics, therapeutics and theranostics.

## 2. Generation of Aptamers by Cell-SELEX

Aptamers are generated by a process called Systematic Evolution of Ligands by Exponential Enrichment (SELEX), first reported in 1990 [11,12]. A variety of targets, including metal ions, small organic molecules, viruses, parasites, tissues and bacterial cells [2,3,4,5], can be utilized for aptamer selection [13]. More importantly, intact cells, especially diseased cells, can act as targets in SELEX [14,15]. In addition, other SELEX methods, such as in vivo SELEX [16], tissue SELEX [17], and 3D cell-SELEX [18], have also been developed. Many techniques, such as surface plasmon resonance [19], robotic SELEX [20], capillary electrophoresis [21] and microfluidics [22] have been used for aptamer selection. 

**Figure 1:**
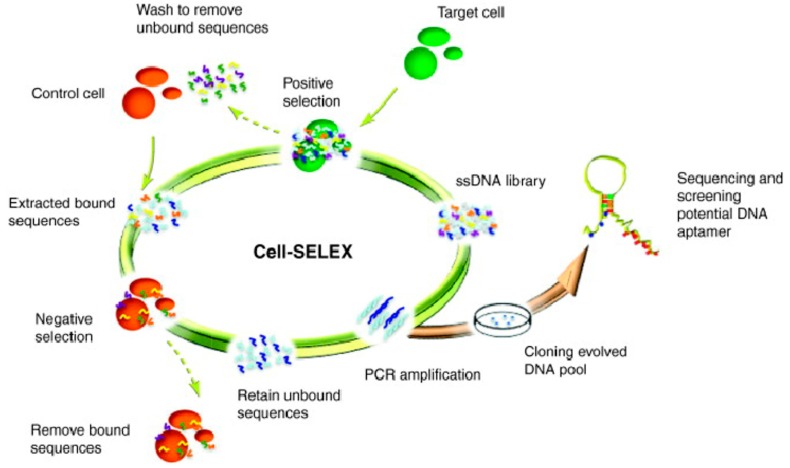
Schematics of cell-based aptamer selection.

The cell-SELEX process (Figure 1) commonly begins with a large random library of about 1 × 10^13^~1 × 10^16^ ssDNA or ssRNA molecules. These molecules are repeatedly incubated with two types of cells, including specific cancer cell types, as the targets, and normal cells, or other types of cancer cells, as counter controls. These molecules undergo repeated interaction with the target of interest, progressively winnowing out non-binders, thus making it possible to obtain aptamer probes which specifically bind only to the targeted cancer cells, while molecules with nonspecific binding to the counter control cells are removed. These enriched molecules are amplified by polymerase chain reaction (PCR). The survivors are sequenced and used as molecular probes [23].

However, some obstacles complicate this process. In particular, aptamers are known to have poor nuclease resistance, although such degradation can be avoided by introducing nucleotide substitution on the 5′-α-P-site or on the 2′-carbon of ribose or by using artificial riboses in the modified library to enhance nuclease resistance [24]. Moreover, natural oligonucleotides have low information density, which can limit functionality and binding affinity. This drawback can be addressed by adding functional groups [25,26,27] or artificially expanded nucleobases [28,29,30] to mimic amino acid side chains in DNA/RNA.

Based on the advantages and technical advances noted above, unprecedented opportunities for biomarker discovery lie ahead for aptamers, in particular for cancer theranostics based on specific recognition. In fact, using cell-SELEX, a number of aptamers for cancer cells have already been selected [31], including breast cancer cells [32], lung cancer cells [33], and leukemia cells [15,34].

## 3. Aptamer-Based Applications

### 3.1. Imaging

The use of aptamers as in vivo imaging probes was introduced in 1997 [35] with a better signal-to-noise ratio compared with the corresponding antibody counterparts. Aptamers have no inherent imaging capacity, thus necessitating conjugation with functional groups or agents with imaging properties, such as fluorophores, iron oxide nanoparticles or other nanoparticles [36]. Fortunately, aptamers can be easily chemically modified, and researchers have developed a wide array of functionalized aptamers for in vivo molecular imaging.

**Figure 2:**
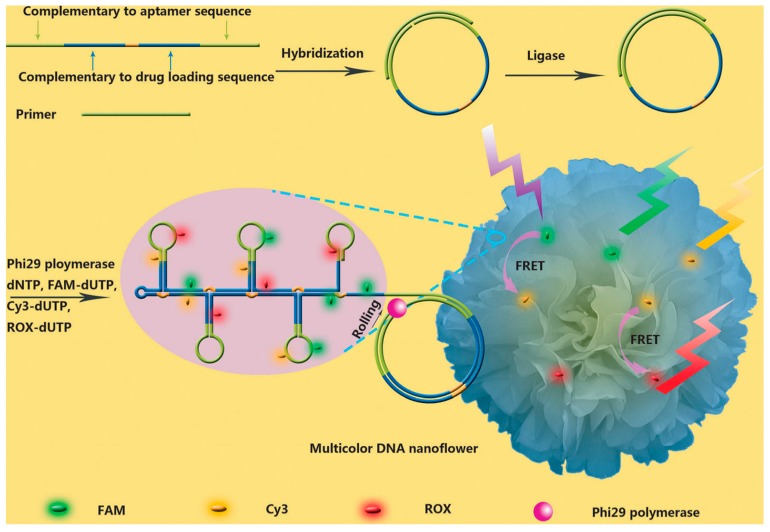
Schematic illustration of aptamer-conjugated DNA nanoflowers (NFs) by rolling circle replication reaction (RCA) to realize cellular imaging with fluorescent resonance energy transfer (FRET) for tracking DOX drug. Reprinted with permission from © (2014) WILEY-VCH Verlag GmbH & Co. KGaA [38].

Fluorophores can be modified on the end of an aptamer through phosphoramidite chemistry. The aptamers will then emit fluorescence for in vivo imaging when the needed photons are provided. The signal for a single aptamer is low, but it will increase when the aptamers circulate and accumulate after reaching the desired target. Further, multiple dyes can be labeled on a given aptamer via chemical reactions or interactions, such as biotin and streptavidin pairing [37], to further amplify fluorescence signaling, enabling easy and rapid identification of the target cells for diagnosis. Hu et al. [38] reported DNA nanoflowers (NFs) conjugated with a fluorescent resonance energy transfer aptamer by rolling circle replication reaction (RCA) to realize cellular imaging and cancer-targeted treatment (Figure 2). NFs covalently modified with three dye molecules generated multifluorescence emissions by a single-wavelength excitation.

**Figure 3:**
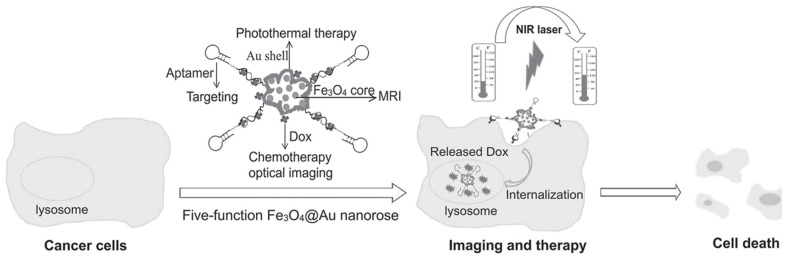
Schematic illustration of Fe3O4@Au nanoroses for cancer cell targeting, MRI, and optical imaging, as well as photothermal and chemotherapy. Reprinted with permission from © (2014) WILEY-VCH Verlag GmbH & Co. KGaA [40].

Aptamers can be conjugated to paramagnetic molecules, such as super-paramagnetic iron oxide nanoparticles (SPIONs) and paramagnetic metal chelates [39], to realize magnetic resonance-based molecular imaging. Li et al. reported the use of aptamer-conjugated Au-coated Fe_3_O_4_ nanoroses for selective and efficient imaging, diagnosis and therapy (Figure 3) [40]. Fe_3_O_4_ nanoroses served as a magnetic resonance imaging (MRI) agent, which could probe variable relaxation rates of protons or certain other nuclei. The relaxation rates vary with nanoparticle interactions, such as aggregation, and can be distinguished by MRI with endogenous contrast.

**Figure 4:**
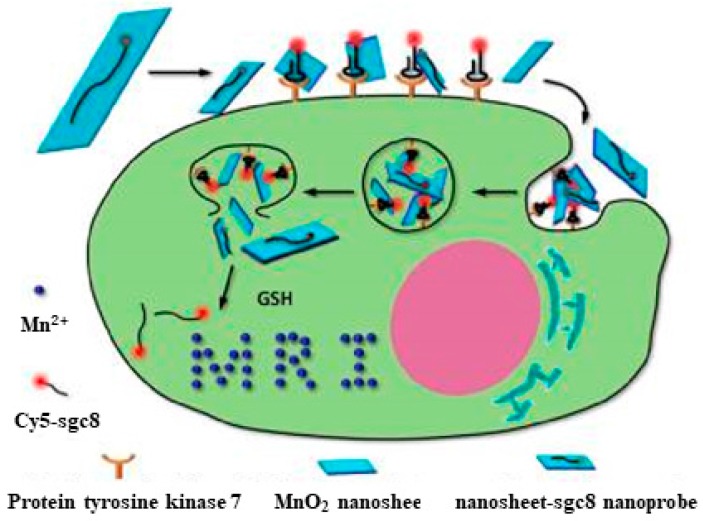
Activation mechanism of the MnO_2_ nanosheet-aptamer nanoprobe for fluorescence/MRI bimodal tumor cell imaging. Reprinted with permission from © (2014) American Chemical Society [42].

To make aptamers effective for in vivo imaging, nanoparticles, or DNA-based nanostructures, can be combined with aptamers to gain greater signal, as well as deeper tissue penetration. Kuai et al. [41] developed circular bivalent aptamers with imaging capability to selectively target cancer cells. Zhao et al. designed a dual-activatable fluorescence/MRI bimodal platform based on a redox-active manganese dioxide (MnO_2_) nanosheet aptamer nanoprobe for targeting cancer cells (Figure 4) [42]. The enhancement in fluorescence signals facilitated in vivo imaging through the reduction of MnO_2_ by GSH and resulting generation of large amounts of Mn^2+^ ions.

### 3.2. Targeted Drug Delivery

Aptamers conjugated with delivery vector systems, such as liposome vesicles, have attracted attention for targeted intracellular drug delivery [43,44]. Most existing aptamers cannot directly migrate through the cell membrane, which calls for the development of new aptamer probes able to penetrate cells for drug delivery. Up to now, the aptamer with most frequent clinical use has targeted prostate-specific membrane antigen (PSMA) [43,45,46]. The Tan group also developed aptamers Sgc8 and TDO5 which act as recognition molecules to bind CCRF-CEM and Ramos cells, respectively. In particular, aptamer Sgc8 is strongly internalized by CEM cells [15] and accumulates in the endosomes. Furthermore, Sgc8 exhibits low cytotoxicity to CEM cells, suggesting that these aptamers can be functionalized for cell type-specific intracellular delivery. Different modes, such as covalent crosslinking, intercalation and self-assembly, can be used to load the drug.

**Figure 5:**
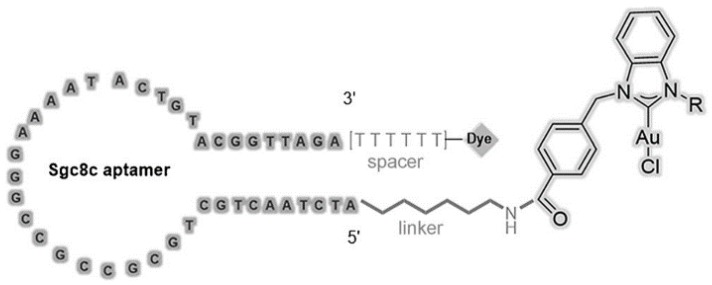
Schematic illustration of aptamer-NHC-Au1 conjugate. Reprinted with permission from © (2016) WILEY-VCH Verlag GmbH & Co. KGaA [48].

**Figure 6:**
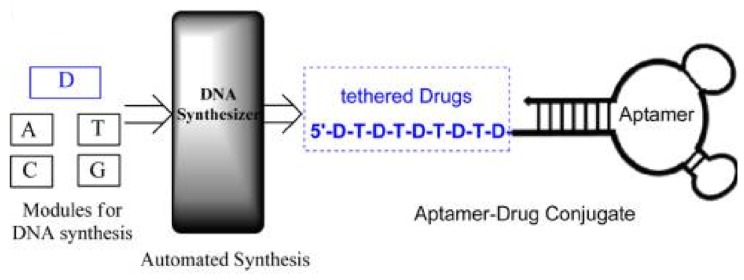
Schematic illustration of automated and modular chemical synthesis of aptamer-drug conjugates (ApDCs) from phosphoramidites A, T, C, G and D. Reprinted with permission from © (2014) American Chemical Society [49].

For instance, covalent crosslinking can be used for aptamer-based drug delivery owing to the facile functionalization of aptamers [47]. Niu et al. presented an approach based on the covalent binding of a cytotoxic N-heterocyclic carbene (NHC)-Au complex to a DNA aptamer. The experimental results showed clear evidence that the NHC-Au^1^-aptamer conjugate was efficient and selective for delivery of doxorubicin (Dox) into CCRF-CEM leukemia cells (Figure 5) [48]. Wang et al. designed aptamer-drug conjugates (ApDCs) for targeted drug delivery with reduced toxicity (Figure 6) [49]. They realized automated and modular synthesis of ApDCs and efficiently incorporated multiple drugs into ApDCs at predesigned positions by a solid-phase therapeutic module for targeted drug delivery. Zhu et al. [50] coupled photosensitizer, single-walled carbon nanotubes (SWNTs) and ssDNA aptamer to form a molecular beacon structure for controllable generation of singlet oxygen (^1^O_2_). The produced ^1^O_2_ could selectively kill SK-BR-3 breast cancer cells, not normal cells. This method should gain wide acceptance as an alternative noninvasive treatment for cancer.

**Figure 7:**
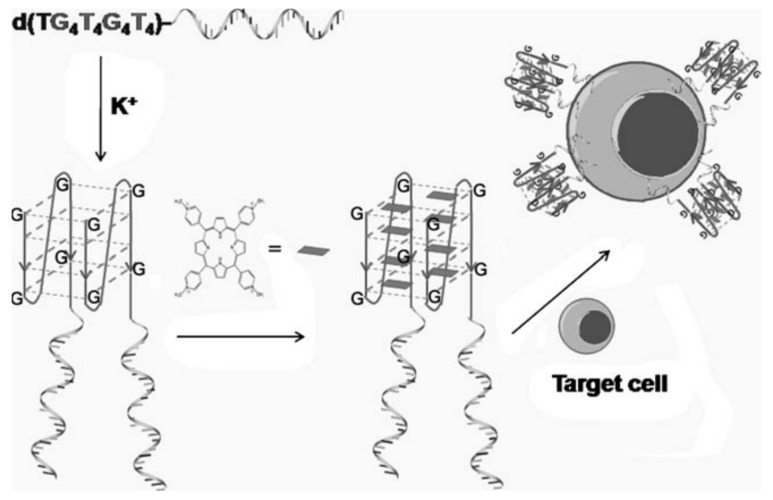
Schematic illustration of a G-quadruplex-aptamer. TMPyP4 is delivered with the G-quadruplex as the drug carrier and the aptamer as the targeting molecule. The helical strand is the aptamer. Reprinted with permission from © (2011) WILEY-VCH Verlag GmbH & Co. KGaA [51].

In addition to covalent crosslinking, intercalation is another efficient drug-loading method for cancer therapy. Double-stranded sequences rich in CG or GC base pairs can intercalate the anticancer drug Dox. With these CG or GC sequences present in the aptamer sequence, internalization can be achieved with decreased cytotoxicity of Dox drug in nontarget cells. Wang et al. combined two functional DNA groups to form a G-quadruplex-aptamer-drug platform by intercalation. One group can recognize the target cells and another can act as a drug delivery carrier for TMPyP4 (Figure 7) [51], which binds and stabilizes the G-quadruplex. Zhu et al. developed aptamer-based DNA nanotrains by hybridization chain reaction (HCR) and utilized them to carry anticancer drugs for cancer therapy [52]. Two molecular beacon-structured DNA sequences self-assembled into tandem “boxcars” were used as carriers to transport the drugs into target cells with no indication of selective cytotoxicity.

Other researchers [53,54,55,56,57] have combined aptamer-based mesoporous nanomaterials as nanotools to effectively enhance drug-carrying capacity. The transported drug could be released by the reduced size of the single-stranded neck region. The strategy is applicable to the transport of anticancer drugs, such as paclitaxel, DOX and camptothecin, with highly efficient delivery.

### 3.3. Therapy

The aim of molecular medicine is evidence-based therapy, i.e., the adoption of a treatment based on cognition of molecular change. Targeted therapy can enhance the curative effect at the molecular level owing to lower cytotoxicity, fewer side effects and higher efficacy. Up to now, some targeting components, such as peptide-, protein- or immunoconjugates, have been recruited to improve the efficiency for targeted cancer therapy [44,58]. Moreover, the Tan group has significantly improved the design of targeted therapy regimens, including targeted phototherapy, gene therapy and chemotherapy, by taking advantage of cell-SELEX to produce cell-specific aptamers having multiple chemical properties against different cancers.

#### 3.3.1. Phototherapy 

**Figure 8:**
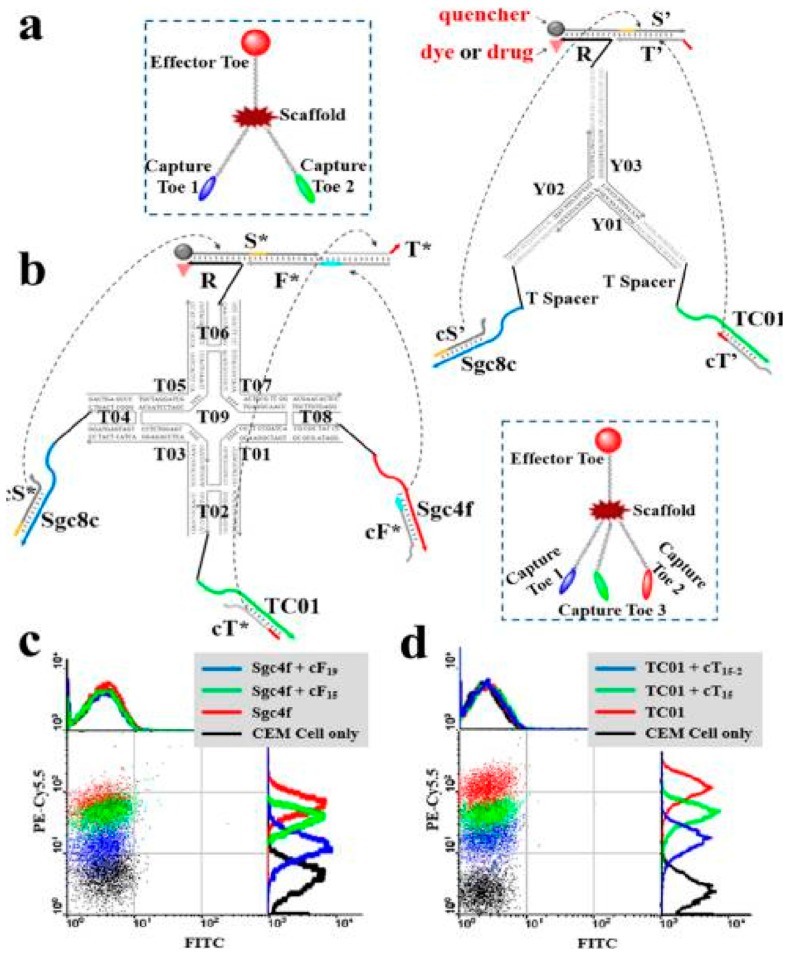
Schematic illustration of modular logic gates assembly for targeted photodynamic therapy. (**a**) Two-input trivalent “Y”-shaped Nano-Claw and (**b**) Three-input tetravalent “X”-shaped Nano-Claw. (**c**,**d**) Flow cytometry experiments to determine the best cDNA sequences with high Cy5.5 fluorescence signal (from biotin-labeled TC01, Sgc4f or Sgc8c aptamer) and low FITC fluorescence signal (labeled on the candidate strands). Reprinted with permission from © (2014) American Chemical Society [61].

**Figure 9:**
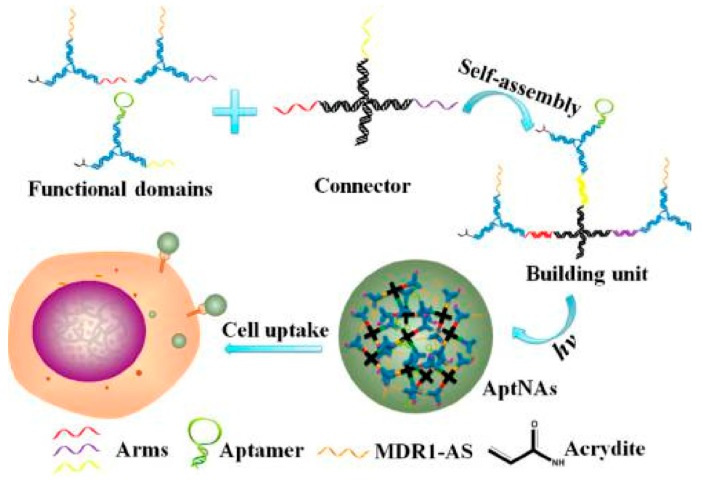
Schematic illustration of multifunctional self-assembled building units and photocrosslinked nanoassembly structure for targeted cancer phototherapy. Multifunctional DNA sequences, including aptamers, acrydite-modified single-stranded DNA, and antisense oligonucleotides are self-assembled to form Y-shaped functional domains, which further link via X-shaped connectors to form building units through the complementary arm sequences. Reprinted with permission from © (2013) American Chemical Society [62].

Recently, photodynamic therapy (PDT), which makes use of a photosensitizer to generate reactive oxygen species under irradiation, has attracted attention in cancer therapy [59,60]. You et al. developed a DNA-based device capable of performing autonomous logic-based analysis of two or three cancer cell-surface markers for cancer therapy (Figure 8) [61]. Multicellular marker-based cancer analysis was based on modular AND, OR and NOT Boolean logic gates using the specific target-recognition properties of DNA aptamers with toehold-mediated strand displacement reactions. A general method was developed for assembling these modular logic gates to execute programmable and higher-order profiling of multiple coexisting cell-surface markers with the capacity to report a diagnostic signal and/or deliver targeted PDT. Wu et al. designed a multifunctional aptamer-based DNA nanoassembly (AptNA) for potential targeted cancer phototherapy (Figure 9) [62].

Many DNA-based building units with 5′-modification of acrydite groups were further photocrosslinked into an aptamer-based nanoassembly structure to realize selective recognition and transport with high programmability, and excellent biostability and biocompatibility. The Tan group also developed other strategies for photothermal therapy based on nanomaterial-aptamer conjugates [63,64].

#### 3.3.2. Gene Therapy

**Figure 10:**
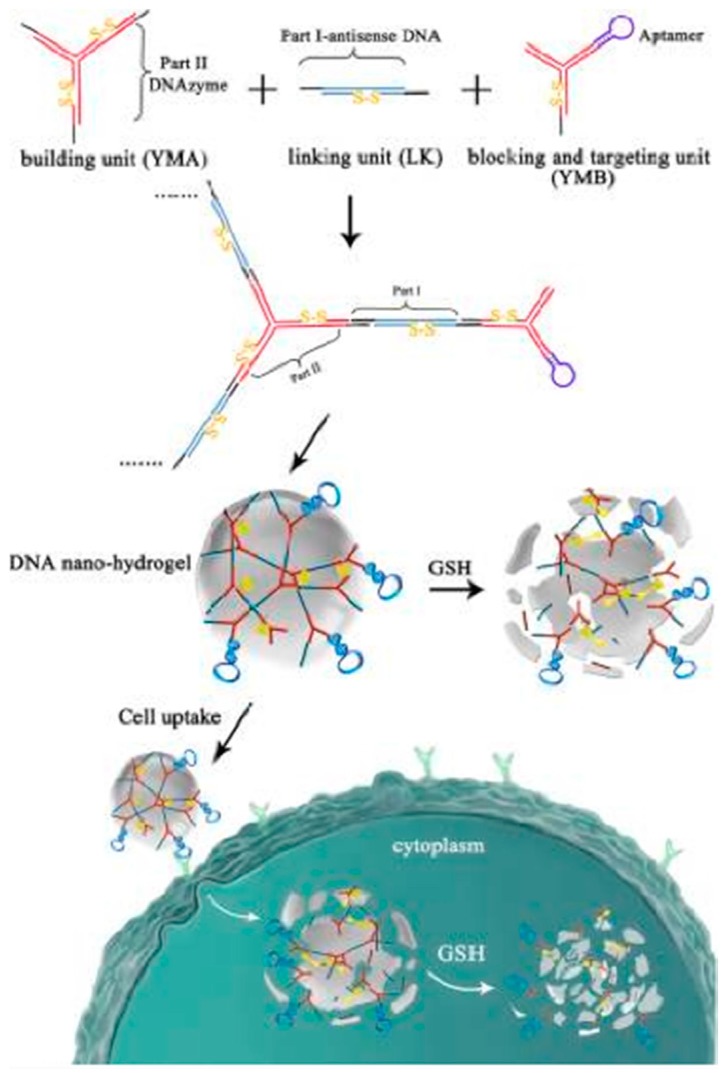
Schematic illustration of DNA nanohydrogels incorporating aptamers for effective targeted gene therapy. Reprinted with permission from © (2015) American Chemical Society [72].

**Figure 11:**
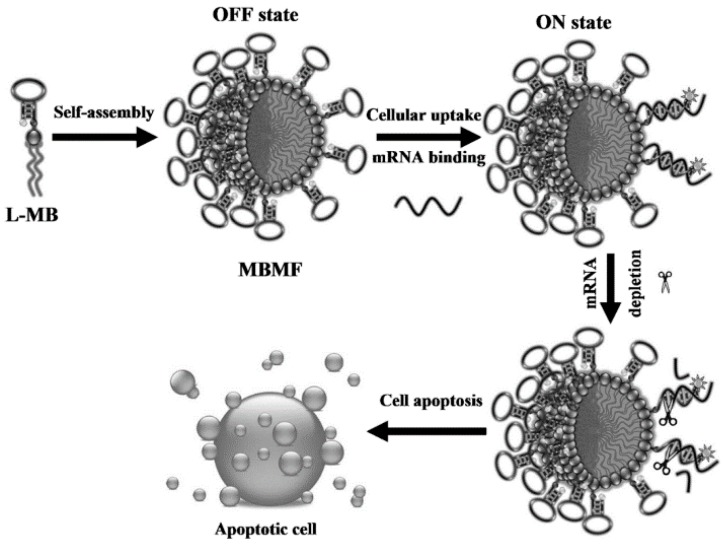
Schematic illustration of molecular beacon micelle flares (MBMFs) for gene therapy. Diacyl-lipid-molecular-beacon conjugates (L-MBs) self-assemble into MBMFs and enter living cells. Before binding their target mRNA, the fluorophore and the quencher of the MBMFs are in close proximity (OFF state). Hybridization between the loop region and the target mRNA separates the fluorophore and the quencher, producing a fluorescence signal (ON state) and a DNA/RNA heteroduplex for RNase H action. Reprinted with permission from © (2013) WILEY-VCH Verlag GmbH & Co. KGaA [73].

Genetic therapy has been introduced into somatic tissues to treat many diseases, including cancer, using antisense oligonucleotides, plasmid DNA, or RNA interference [65,66,67,68,69]. DNA fragments can hybridize with these complementary sequences, e.g., the loop portion of a molecular beacon structure [70,71], which greatly facilitates the improvement of gene therapy or disease diagnosis. Li et al. reported size-controllable and stimuli-responsive DNA nanohydrogels, incorporating aptamers as effective targeted gene therapy vectors (Figure 10) [72]. DNA was used for gene therapy vectors constructed through a self-assembly process using three kinds of building units. Through this facile modular assembly, these aptamer-based DNA nanohydrogels hold great promise for targeted gene therapy with superior biocompatibility and efficient cellular uptake. Chen et al. designed a sensitive and selective approach based on molecular beacon micelle flares (MBMF) for mRNA detection and gene therapy (Figure 11) [73]. MBMF hybridization can lead to apoptosis of cancer cells by inducing gene silencing.

#### 3.3.3. Chemotherapy

Enhancement of chemotherapy efficacy and comprehensive toxicity reduction can be achieved through conjugation of anticancer agents to targeting antibodies and aptamers [74,75]. Wang et al. [49] utilized solid-phase synthesis technology to generate DNA from individual phosphoramidite building blocks, namely, A, T, C and G, which can be used for automated and sequence-predesigned DNA synthesis. Using this technique, next-generation aptamer-drug conjugates were designed for targeted chemotherapy. 

**Figure 12:**
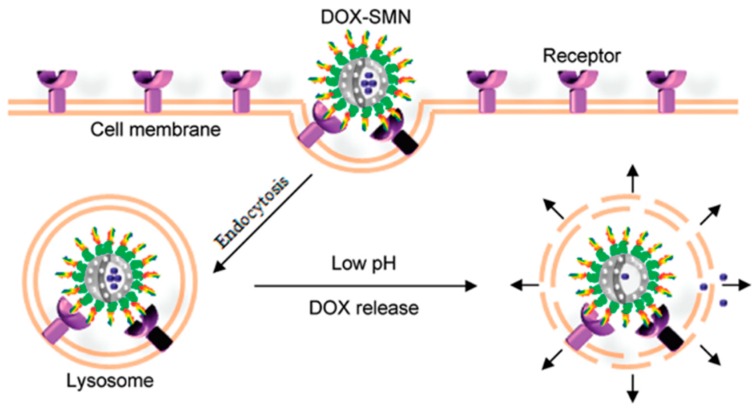
Schematic illustration of SMNs for targeted cancer chemotherapy. Owing to surface coating of aptamers, DOX-SMNs specifically enter target cancer cells through receptor-mediated endocytosis and reside in acidic lysosomes. Reprinted with permission from Reference [76]. © (2011) American Chemical Society.

Chen et al. reported a smart multifunctional nanostructure (SMN) constructed from a porous hollow magnetite nanoparticle (PHMNP), a heterobifunctional PEG ligand and an aptamer to achieve targeted chemotherapy and magnetic resonance imaging of cancer cells in vitro (Figure 12) [76]. The PHMNPs were synthesized by a three-step reaction, including functionalization with PEG ligands and modification with DOX. Targeting aptamers were then introduced by reaction with the PEG ligands. Huang et al. [77] linked DNA aptamer Sgc8c to the anticancer agent Dox via covalent bonding. Compared with other reported ineffective Dox-conjugates, strategies based on the Sgc8c-Dox conjugate endowed targeted chemotherapy with enhanced feasibility, multiple potency and broad implications for targeted chemotherapy [74].

## 4. Biomarker Discovery for Cancers

According to the definition from the National Institutes of Health [78], a biomarker is “a characteristic that is objectively measured and elevated as an indicator of normal biological processes, pathogenic processes or pharmacologic response to a therapeutic intervention”. For cancer, the definition narrows to “a biological molecule found in blood, other body fluids, or tissues that is a sign of a normal or an abnormal process, or of a condition or a disease such as cancer” [79]. Thus, a biomarker can be applied to prognosis or diagnosis in a clinical setting. It can also be applied to either monitor disease progression or identify possible biochemical origins, achieving both by an immortalized cell line related to cancers, essentially because a biomarker molecule can differentiate “normal” from “abnormal” cells. Although some molecular markers have been established, such as nucleic acids, serum proteins, peptides, antibodies, tissues, biological fluids or small molecules (sugars, steroids or any other molecule present in cells) [80], biomarker discovery remains an urgent challenge in the field of molecular medicine [81,82], especially for cancers, because early diagnosis and effective therapy are greatly impeded by the lack of discriminatory biomarkers.

What mainly limits the current progress of biomarker discovery is under-representation of membrane proteins in proteome analysis. In addition, membrane proteins account for 30% of the proteins in the body, but fewer than 5% of membrane proteins can be recognized using two-dimensional gel electrophoresis combined with mass spectrometry (2D-GE-MS) [83]. However, differential selection of aptamers based on cell-SELEX promotes discovery of membrane protein biomarkers [81], as described in the following sections.

**Figure 13:**
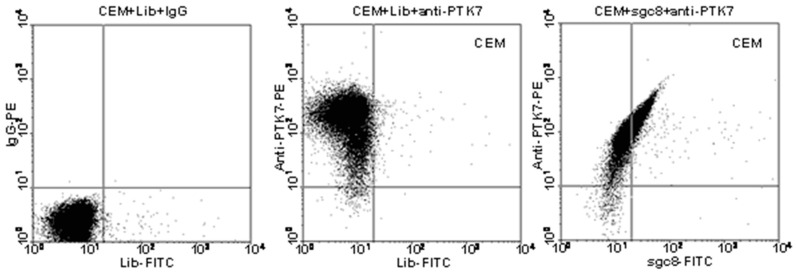
Flow cytometry analysis of CEM cells targeted with anti-PTK7-PE or sgc8-FITC. Reprinted with permission from © (2009) American Chemical Society [94].

In exploratory work to establish cell-SELEX, the Tan group found the biomarker protein for target CEM cells by using highly specific aptamer Sgc8 [84]. A library of 10^15^ random DNA sequences was incubated with T-ALL cells (CCRF-CEM) using Ramos cells for counter selection. After selecting Sgc8 as a promising aptamer candidate, the target membrane protein was then identified. First, a target cell lysate was prepared, and the membrane protein fraction was separated from the total soluble proteins by a membrane protein extraction kit according to the manufacturer’s instructions. Then biotinylated Sgc8 aptamer and streptavidin magnetic beads were used to capture and isolate the membrane proteins having high affinity to aptamer. It was observed by flow cytometry that anti-protein tyrosine kinase 7 (PTK7) antibody and Sgc8 co-bind, rather than competitively bind, with the transmembrane protein PTK7 (Figure 13). Subsequently, the target membrane protein was separated using sodium dodecyl sulfate-polyacrylamide gel electrophoresis (SDS-PAGE) and sequenced by liquid chromatography-mass spectrometry (LC-MS). After confirmatory experiments, PTK7, a transmembrane receptor commonly found on cancer cells, was finally identified and verified as the target biomarker [84]. These results indicated a relationship between T-ALL cancer cells and overexpression of PTK7. Aptamer Sgc8 is now used as a molecular recognition probe with various nanomaterials in a wide range of bioapplications, including tumor imaging [42,85,86], cancer therapy [48,62,87,88], targeted delivery [49,52,89,90], and cancer detection [61,91].

**Figure 14:**
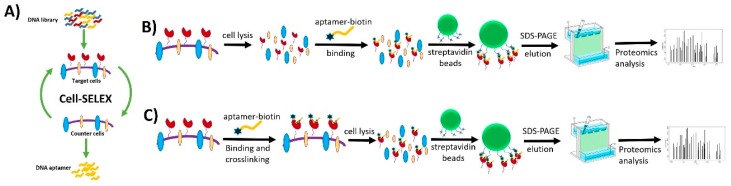
Aptamer-based isolation of target protein biomarkers with crosslinking aptamers and target proteins for cancer cell surface biomarker discovery. (**A**) Cell-SELEX technology selects a panel of aptamers capable of distinguishing molecular differences between two types of cells. (**B**) Aptamer-assisted affinity purification without cross-linking aptamers and target proteins as targets proteomics strategy for cancer cell surface biomarker discovery. (**C**) Aptamer-assisted affinity purification with cross-linking aptamers and target proteins as targeted proteomics strategy for cancer cell surface biomarker discovery. Reprinted with permission from © (2017) American Chemical Society [95].

The same biomarker elucidation process was used with aptamer TDO5 to identify the immunoglobulin heavy mu chain (IGHM) as a biomarker for B-cell Burkitt’s lymphoma cell line [92]. In that selection case, UV-induced group 5-iododeoxyuridine (5-dUI) was covalently linked with biotinylated aptamer TDO5 by replacing several deoxythymidines (dTs). The overexpressed immunoglobulin heavy mu chain (IGHM) on premature B-lymphocytes was determined to be the target of TDO5 aptamer with close correlation to Burkitt’s lymphoma research [93,94]. The target Ramos cells were lysed, and aptamer-conjugated proteins were isolated by streptavidin beads, followed by cleavage of the disulfide bonds in the captured aptamer-protein complex to release the target. The results of LC-MS and SDS-PAGE also demonstrated the target protein to be B-cell receptor IGHM [94]. Utilizing this covalent linkage process, the capture, enrichment and aptamer-based isolation of target protein receptors have been improved (Figure 14) [95].

**Figure 15:**
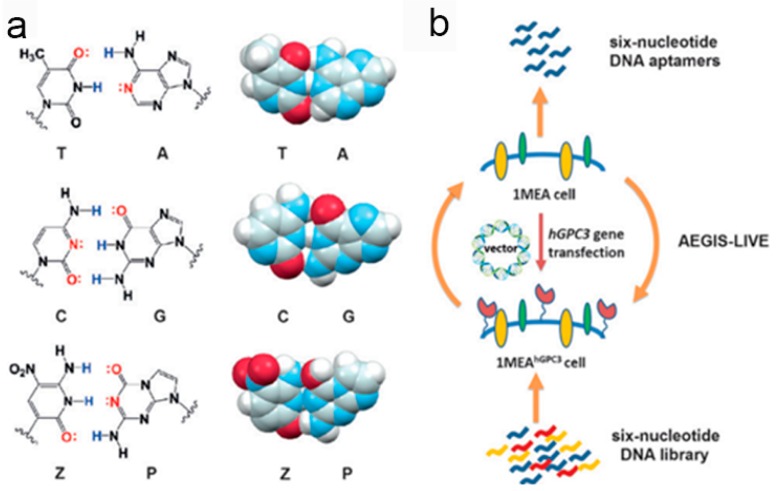
Schematic illustration of hybridization of the six nucleotides for an artificially expanded genetic information system (AEGIS) in Laboratory In Vitro Evolution (LIVE). (**a**) Molecular structures and space-filling models of C:G, T:A, and Z:P pairs showing their similarity; (**b**) Application of the artificial aptamers in biomarker discovery based on the AEGIS-LIVE selection procedure. Reprinted with permission from © (2016) WILEY-VCH Verlag GmbH & Co. KGaA [96].

According to Benner, et al. [96], if “the Watson-Crick model supported an enlarged molecular system and an enlarged molecular biology,” then an artificially expanded genetic information system, such as that described by Zhang, et al. [95], should bring “enlarged functional potential,” with concomitant implications for biomarker discovery. More specifically, in their expanded system for SELEX biomarker discovery, Zhang, et al. utilized an artificial base pair to select aptamers against living cells [28,29] and cell-surface target proteins [96] (Figure 15). In this research, a pool of six-nucleotide aptamers was used to target cells with specific overexpression of glypican 3 protein (GPC3) on the cell surface, along with counterselection by laboratory in vitro evolution (LIVE). The selection-counterselection strategy starts with a known biomarker protein, which means the aptamer recognition biology is already well established. Implantation of these two artificial bases improved the functionality of the DNA library pool and tremendously enhanced information feedback from the conjugated complex. Also, based on negative selection, this new SELEX technology with expanded artificial bases virtually guarantees the removal of off-target binding DNAs.

**Figure 16:**
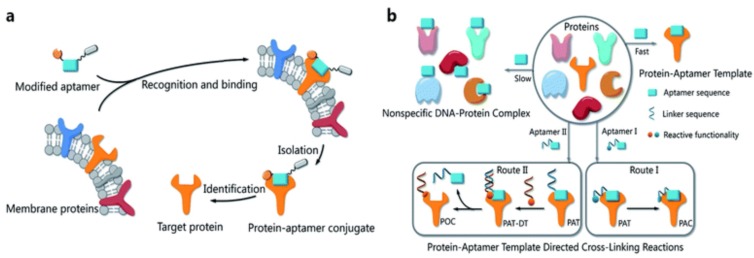
Schematic illustration of (**a**) one-step bioorthogonal reaction for biomarker discovery; (**b**) PAT-directed reactions for the preparation of protein-aptamer conjugates (PAC, route I) or protein-oligonucleotide conjugates (POC, route II). Reprinted with permission from © (2016) Royal Society of Chemistry [97].

Very recently, Wang et al. exploited the bioorthogonal interaction between protein and aptamer for biomarker discovery. They selectively conjugated DNA aptamers with a protein via a protein-aptamer template (PAT)-directed reaction because interactions between protein and aptamer are bioorthogonal (Figure 16) [97]. A modified aptamer library reservoir was used in the PAT-directed reactions, and an *α*,*α*-*gem*-difluoromethyl carboxyl group (F-carboxyl) was found to be suitable for selective and site-specific combination and functionalization. This approach of conjugating the aptamer to ascertain the interaction location, i.e., the protein, on the cell surface is promising for oriented modification of functional molecules and groups in biomedical and biological research.

A superior strategy called “parallel mode” was introduced by Shangguan and Wang et al. using a formaldehyde crosslinking concept [98]. Two aptamers, Sgc3b and Sgc4e, were successfully utilized to identify the corresponding biomarker protein selectin L and integrin α4 of Jurkat cells. A fluorophore was linked with the aptamer to track and optimize the binding by flow cytometry analysis. The authors adopted a quantitative proteomic method by labeling a stable isotope on amino acids when culturing Jurkat cells, and they utilized crosslinked, aptamer-based affinity purification by a streptavidin agarose resin. Finally, the target proteins were identified via SDS-PAGE and LC-MS/MS after heating for 1 h at 95 °C. They recommended this method for elucidation of general protein targets.

In another example, Yang et al. reported an adhesion molecule biomarker, epithelial cell adhesion molecule (EpCAM), on the surfaces of epithelial cells (circulating tumor cells (CTCs)) [99]. In the SELEX process, recombinant EpCAM was labeled by His-Tag, and this complex served as target. Random DNA aptamers were delivered to target EpCAM protein and EpCAM-expressing cell lines. Subsequently, aptamer SYL3C was verified and is now broadly applied for cell-related research [87,100,101]. 

Recently, Yarden et al. developed a 14-nt aptamer to target a biomarker for native human epidermal growth factor receptor 2 (HER2) protein [102]. HER2 protein is a member of the rbB family of receptor tyrosine kinases overexpressed in many tumors that can be targeted to develop anticancer drugs. The results showed that the selected aptamer-based trimer could be effectively delivered to inhibit the growth of tumor cells and that the efficacy was better by almost two-fold compared to delivery by anti-HER2 mAb. The mechanism indicated that the conjugation of aptamer with HER2 receptor could induce cellular internalization, cytoplasmic translocation, and lysosomal degradation to suppress tumor growth.

Many other researchers have also devoted themselves to SELEX and biomarker discovery-related studies. Mayer et al. exploited a click-chemistry approach to functionalize the oligonucleotide library for further versatility [103]. Bertozzi et al. induced a proximity-increased bioorthogonal ligation strategy for crosslinkage between azidosugar-labeled glycoproteins and an aptamer conjugated with cyclooctyne [104]. Vinkenborg et al. developed a strategy for aptamer-based affinity labeling of protein using three aptamers with different structures and a phenylazide as photocrosslinker [105]. Kimoto et al. reported a method to enhance affinity for biomarker discovery by adding a fifth nucleotide with a hydrophilic functional group to the aptamer library [30].

The aforementioned works indicate that the cell-SELEX technique and its numerous iterations and variations will only accelerate the development of biomarker discovery. They also implicate that the selected aptamers have potential for alternative theranostic applications by specific recognition of target biomarkers. All these efforts continuously improve the progress in biomarker discovery by focusing on cell-surface proteins, and they will, as a result, significantly contribute to the broad development of personalized medicine.

## 5. Conclusions and Perspectives

SELEX technologies and aptamers have been studied for more than a quarter century since first reported, and applications have been developed to meet the needs of molecular-level personalized medicine. Many kinds of aptamers have been successfully selected and continuously applied in multiple biotechnical fields and clinical practice, such as imaging, drug delivery, therapies and biomarker discovery. However, some difficulties still remain, despite the great progress in aptamer and aptamer-based cancer biomarker discovery, and applications are still in an early stage. Moreover, the applications in commercial areas are limited, although aptamers are widely studied in many fields. First, for cell-SELEX, the aptamer selection process usually takes more than 30 days, while the results still display some degree of randomness, which blocks the improvement of aptamer-based biomarker discovery in some aspects. Successful aptamer selection will be evaluated by the elimination of phenotypic heterogeneity from different cell passages and shortened turn-around time, with ideally just one round needed to precisely select effective aptamers. Second, it is time-consuming to identify aptamers specific for different cancer-related samples, which are necessary for the discovery of more cancer biomarkers. Third, binding affinity enhancement between aptamers and their targets is still urgently needed to avoid off-target recognition and to improve protein extraction efficiency from complex samples (e.g., the cross-linking methods). The fourth concern is the natural self-disadvantages of DNA/RNA molecules as binding moieties. Very limited folding patterns can be produced, because, with only A-T and C-G building blocks, the possible information density is low. Moreover, the interactions by which the present bases establish DNA/RNA molecules include only hydrogen bonding between nucleobases, aromatic ring stacking among nucleobases and electrostatic interactions. Another current major obstacle for aptamer applications is the need for enhancement and optimization of therapeutic efficacy, stability and half-life within a patient, especially in complex microenvironments surrounding disease tissue. To solve this, “locked DNAs” may be one way to improve the stability. In addition, the Tan group has successfully synthesized artificial DNA bases to endow aptamers with additional innovative properties to provide even more innovative ways to discover new effective biomarkers, efficient therapeutic pathways, or approaches for cellular uptake. Continued research will lead to improved understanding of cancer at the molecular level. This improvement will further inspire exciting new technologies for imaging, drug delivery, cancer therapy and cancer biomarker discovery, leading to feasible and realizable personalized treatment methods.
